# Three-Dimensional Sound Source Localization with Microphone Array Combining Spatial Entropy Quantification and Machine Learning Correction

**DOI:** 10.3390/e27090942

**Published:** 2025-09-09

**Authors:** Guangneng Li, Feiyu Zhao, Wei Tian, Tong Yang

**Affiliations:** 1 College of Computer Science, South-Central Minzu University, Wuhan 430071, China; gnli19@126.com (G.L.); victor-tian888@163.com (W.T.); 2024120469@mail.scuec.edu.cn (T.Y.); 2 Hubei Digital Manufacturing Key Laboratory, Wuhan University of Technology, Wuhan 430074, China

**Keywords:** acoustic detection, sound source localization (SSL), spatial entropy, machine learning

## Abstract

In recent years, with the popularization of intelligent scene monitoring, sound source localization (SSL) has become a major means for indoor monitoring and target positioning. However, existing sound source localization solutions are difficult to extend to multi-source and three-dimensional scenarios. To address this, this paper proposes a three-dimensional sound source localization technology based on eight microphones. Specifically, the method employs a rectangular eight-microphone array and captures Direction-of-Arrival (DOA) information via the direct path relative transfer function (DP-RTF). It introduces spatial entropy to quantify the uncertainty caused by the exponentially growing DOA combinations as the number of sound sources increases, while further reducing the spatial entropy of sound source localization through geometric intersection. This solves the problem that traditional sound source localization methods cannot be applied to multi-source and three-dimensional scenarios. On the other hand, machine learning is used to eliminate coordinate deviations caused by DOA estimation errors of the direct path relative transfer function (DP-RTF) and deviations in microphone geometric parameters. Both simulation experiments and real-scene experiments show that the positioning error of the proposed method in three-dimensional scenarios is about 10.0 cm.

## 1. Introduction

Accurate positioning is usually achieved through electronic device transmissions, such as global positioning system’s (GPS’s) National Marine Electronics Association (NMEA) [1,2], base station’s multi-in multi-out (MIMO) [3], Wi-Fi’s received signal strength indicator (RSSI) [4,5], and ultra-wideband (UWB) [6]. For indoor environments where obstacles limit the reception of GPS signals or when there are no communication devices available for transmitting messages, sound source localization (SSL) that does not rely on communication systems is considered a highly important technology for replacing electronic devices [7,8]. In industrial applications, sound source detection can be used for fault diagnosis and early warning systems [9,10,11,12]. In human–machine interaction, it is significant in allowing robots to perceive their environment, thus improving their interaction with humans [13,14,15,16]. Furthermore, it can be applied to the analysis of acoustic detection and speech enhancement, among other areas [17,18,19,20]. Therefore, developing accurate and efficient SSL technology is essential for these diverse applications.

Thanks to the invention of many direction-of-arrival (DOA) algorithms, such as generalized cross-correlation (GCC-PHAT) [21,22,23,24,25] and Multiple Signal Classification (MUSIC) [26], DOA-based source localization is currently quite mature. In real-world scenarios, the presence of reverberation can severely affect the estimation of DOA. The direct path relative transfer function (DP-RTF) estimates DOA by calculating the characteristics of the direct path of sound reaching two microphones, thereby improving the accuracy of localization [27]. Li et al. extended this method to the measurement of DOA for multiple sound sources by combining it with the Gaussian Mixture Model (GMM) [28]. Based on DOA, the differentiated positioning requirements have driven research on various microphone array configurations and algorithms. For example, some methods can only provide the DOA information of sound sources [12,24,29,30,31,32], while others can give the 2D coordinates of sound sources [10,11,28,33]; some methods are only applicable to single-sound-source scenarios [21,33,34,35,36], whereas others can achieve sound source localization and output relevant information in both single-sound-source and multi-sound-source scenarios [13,28,37,38]. Positioning technologies that rely on a single dimension or a single sound source can hardly meet practical needs.

In the field of acoustics, especially in the problems of 3D sound source localization and multi-source localization, traditional methods for solving sound source coordinates based on DOA are faced with many challenges. As the number of sound sources increases, the number of DOA combinations between microphones grows exponentially, leading to a sharp expansion of the solution space and a significant increase in uncertainty during the solving process. This phenomenon is highly consistent with the increase in system uncertainty described by information entropy [39], which provides a theoretical basis for introducing information entropy into multi-source localization research. Essentially, entropy is a quantitative representation of the degree of disorder in the distribution of matter, energy, information, or phenomena in space. When this concept is extended to spatial research, it becomes a powerful tool for gaining insights into the characteristics of spatial distribution. In positioning-related studies, spatial entropy has demonstrated unique value in multiple practical scenarios. To reduce long-term positioning drift, Reference [40] introduces an entropy-based invisible position recognition module. Specifically, it judges whether the current position is a new one by calculating the entropy of the output vector; if the entropy of the predicted vector is higher than a threshold, the position is considered a new one. Reference [41] proposes a new entropy-based feature selection method for real-time simultaneous localization and mapping (SLAM) in mobile robot navigation. Based on information entropy theory and data association methods, this method initializes new features into the map, matches measured values with map features, and removes outdated features.

Our proposed method aims to solve the problem of 3D localization in multi-sound-source scenarios: spatial entropy is introduced to quantify the uncertainty caused by the exponentially growing DOA combinations between microphones as the number of sound sources increases; meanwhile, the multi-group DOA features of the microphone array are utilized to reduce the spatial entropy of sound source localization in three-dimensional space, and finally determine the three-dimensional coordinates of the sound source; additionally, machine learning is used to eliminate the sound source coordinate deviations caused by DOA estimation errors, thereby improving localization accuracy. The remaining sections of this article are organized as follows. Section 1 elaborates on the principle of the DP-RTF algorithm for solving DOA, and the process of introducing spatial entropy and reducing it using a microphone array, and introduces the specific scheme of using machine learning to improve positioning accuracy. Section 2 explains the sample collection process in simulation experiments and the analysis of positioning results of the constructed model. Section 3 demonstrates the application process of this method in actual scenarios through an example. Section 4 summarizes the research conclusions and looks forward to future directions.

## 2. Sound Source Localization Based on DOA and an 8-Microphone Array

**Figure 1:**
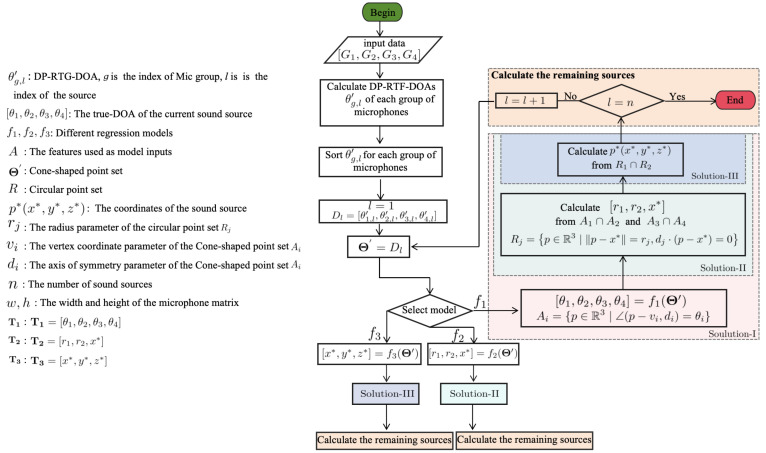
Framework diagram of the proposed method.

We have designed a 3D SSL method using a 4-group, 8-microphone array. We have proven that this method can determine the 3D coordinates of a sound source and demonstrated its feasibility in multi-source scenarios. Our method can be summarized as follows (Figure 1). Firstly, we process the data collected by the four groups of microphones into the DOA based on DP-RTF (DP-RTF-DOA). At the same time, we will also determine the number of potential speakers *n*. Next, we sort the DP-RTF-DOAs of each microphone group so that the DOAs in the same order for each group of microphones belong to the same speaker. Then, we extract the DP-RT-DOAs of each speaker from the four sets of microphones as features $\Theta^{\prime }$, and use different processing solutions to calculate the true coordinates of the sound source based on these features. Based on the characteristics of the microphone array, we designed three solutions combined with regression models to infer the coordinates of the sound sources.

### 2.1. DOA Estimation Based on DP-RTF

In complex acoustic environments, particularly in the presence of noise and reverberation, accurately localizing multiple sound sources presents a challenging task. To overcome these limitations, the DP-RTF has been proposed as a more robust binaural feature. The following introduces the derivation process of the DP-RTF [28,38].

#### 2.1.1. Principle of Single-Source DP-RTF Estimation

In the case of a single source without noise, the received binaural signals are x(t)=s(t)∗a(t) and y(t)=s(t)∗b(t), where s(t) is the non-stationary source signal, and a(t) and b(t) are the binaural room impulse responses. In the STFT domain, to more accurately represent the linear filter with a long impulse response, cross-band filters and the Convolutional Transfer Function (CTF) approximation are introduced. Based on the CTF, x(t) and y(t) in the STFT domain are approximated as

(1) $$x_{p,k}=\sum_{p^{\prime }=0}^{Q-1}s_{p-p^{\prime },k}a_{p^{\prime },k}=s_{p,k}*a_{p,k},$$

(2) $$y_{p,k}=\sum_{p^{\prime }=0}^{Q-1}s_{p-p^{\prime },k}b_{p^{\prime },k}=s_{p,k}*b_{p,k},$$

where $s_{p,k}$ is the STFT coefficient of the source signal at the time–frequency (TF) bin (p,k); *p* and *k* represent the time frame and frequency; $a_{p,k}$ and $b_{p,k}$ are the CTF coefficients of the left and right channels, representing the transfer functions from the source to the microphones; and *Q* is the number of CTF coefficients, related to the reverberation time. The DP-RTF is defined as $\frac{b_{0,k}}{a_{0,k}}$. By processing

(3) $$x_{p,k}*b_{p,k}=y_{p,k}*a_{p,k},$$

we get

(4) $$y_{p,k}=z_{p,k}^{⊤}g_{k},$$

where

(5) $$z_{p,k}={\left[x_{p,k},\cdots ,x_{p-Q+1,k},y_{p-1,k},\cdots ,y_{p-Q+1,k}\right]}^{⊤},$$

(6) $$g_{k}={\left[\frac{b_{0,k}}{a_{0,k}},\cdots ,\frac{b_{Q-1,k}}{a_{0,k}},-\frac{a_{1,k}}{a_{0,k}},\cdots ,-\frac{a_{Q-1,k}}{a_{0,k}}\right]}^{⊤}.$$

Multiplying both sides of $y_{p,k}=z_{p,k}^{⊤}g_{k}$ by $y_{p,k}^{*}$ and taking the expectation, we obtain

(7) $${\hat{\phi }}_{yy}\left(p,k\right)={\hat{\phi }}_{zy}^{⊤}\left(p,k\right)g_{k},$$

where $y_{p,k}^{*}$ is the complex conjugate of $y_{p,k}$. High-speech-power frames are used to construct a linear equation to solve for $g_{k}$, and its first component (denoted as $c_{p,k}$) is the estimated DP-RTF.

#### 2.1.2. Extension of Multi-Source DP-RTF Estimation

In the case of multiple sources, since different frames may correspond to different sources, the single-source DP-RTF estimation method cannot be directly applied. Based on the W-Disjoint Orthogonality (WDO) assumption [42,43], only one source is active in a small TF region, and the DP-RTF is estimated for each TF bin. Considering *O* consecutive frames, the equation

(8) $${\hat{\phi }}_{yy}^{s}\left(p,k\right)={\hat{\Phi }}_{zy}^{s}\left(p,k\right)g_{p,k}$$

is constructed, where

(9) $${\hat{\phi }}_{yy}^{s}\left(p,k\right)={\left[{\hat{\phi }}_{yy}^{s}\left(p-O+1,k\right),\cdots ,{\hat{\phi }}_{yy}^{s}\left(p,k\right)\right]}^{⊤},$$

(10) $${\hat{\Phi }}_{zy}^{s}\left(p,k\right)={\left[{\hat{\phi }}_{zy}^{s}\left(p-O+1,k\right),\cdots ,{\hat{\phi }}_{zy}^{s}\left(p,k\right)\right]}^{⊤},$$

(11) $$e\left(p,k\right)={\left[e\left(p-O+1,k\right),\cdots ,e\left(p,k\right)\right]}^{⊤}.$$

When O≥2Q−1 and e(p,k) is stationary and independent, the optimal estimate of $g_{p,k}$ is

(12) $${\hat{g}}_{p,k}={\left({\hat{\Phi }}_{zy}^{s}{\left(p,k\right)}^{H}{\hat{\Phi }}_{zy}^{s}\left(p,k\right)\right)}^{-1}{\hat{\Phi }}_{zy}^{s}{\left(p,k\right)}^{H}{\hat{\phi }}_{yy}^{s}\left(p,k\right).$$

The value of parameter *O* must satisfy two theoretical constraints: First, the rank condition. When O≥2Q−1, the coefficient matrix of the system of equations is of full rank, ensuring a unique solution for $g_{p,k}$. From this, the theoretical minimum value of O is calculated as 2×12−1=23, and a larger *O* leads to a better effect in canceling reverberation and noise. Second, the computational complexity constraint. Increasing *O* can improve estimation robustness but will increase the computational load; thus, it is necessary to balance accuracy and efficiency on the basis of the theoretical minimum value while considering real-time requirements. By synthesizing the above constraints, *O* = 32 was finally adopted in the experiment.

#### 2.1.3. Multi-Source DOA Estimation

The DP-RTF features $c_{p,k}$ are then clustered using a Complex Gaussian Mixture Model (CGMM), and the speaker directions are estimated by maximizing the following penalized log-likelihood function:

(13) $$\begin{array}{c} \log L\left(C|\alpha \right)=\sum_{k=0}^{K-1}\sum_{p\in P_{k}}\log\left(\sum_{s=1}^{S}\alpha_{s}N_{c}\left(c_{p,k};c_{k}^{s},\sigma^{2}\right)\right)\;-\gamma \alpha^{⊤}\log\left(\alpha \right), \end{array}$$

where $\alpha ={\left[\alpha_{1},\alpha_{2},\ldots ,\alpha_{S}\right]}^{⊤}$ is the prior probability vector of candidate locations, with $\alpha_{s}$ representing the prior probability that a source is located at the *s*-th candidate position. The vector satisfies $\sum_{s=1}^{S}\alpha_{s}=1$. γ is the weight of the entropy penalty term, which controls the trade-off between the log-likelihood and the sparsity of α. A larger γ enforces a sparser solution. $N_{c}\left(c_{p,k};c_{k}^{s},\sigma^{2}\right)$ is the complex Gaussian distribution, representing the probability of the observed binaural feature $c_{p,k}$ given that it is emitted by a source located at the *s*-th candidate position. $\sigma^{2}$ is the variance of the complex Gaussian distribution, assumed to be constant across all frequencies and candidate locations. $c_{k}^{s}$ is the predicted binaural feature at frequency *k* for the *s*-th candidate location, computed from the direct-path propagation model:

(14) $$c_{k}^{s}=\exp\left(-j2\pi \frac{f_{s}\cdot k}{N}\tau_{s}\right)$$

where $f_{s}$ is the sampling rate, $\tau_{s}$ is the time delay at the *s*-th candidate location, and *N* is the length of the Fourier transform.

After obtaining α, the positions of the peaks in α represent the locations of the sound sources, and the number of peaks represents the number of sound sources. Thus, the number of speakers *n* can be determined.

### 2.2. Quantitative Model of Spatial Entropy in 3D Multi-Sound-Source Scenarios

We have devised a localization method utilizing a 4-group, 8-microphone array to determine the 3D coordinates of a sound source in space based on DOA measurements, as illustrated in Figure 3a. Each pair of microphones forms a microphone group, with a spacing of *d* between them, used for DOA measurement. There are four groups of microphones, $G_{1},G_{2},G_{3}$, and $G_{4}$, arranged in a 2 by 2 matrix formation. The rows of the matrix align with the connecting lines of the microphone pairs, while the columns are perpendicular to these lines. The height of the microphone group matrix, *h*, runs parallel to the *x*-axis, and its width, *w*, runs parallel to the *z*-axis.

For the 3D localization scenario with an 8-microphone array proposed in this document, a spatial entropy for sound source localization can be defined to quantify the uncertainty in the solution process. Assume there are *n* sound sources in the scenario, and the set of DOAs measured by 4 groups of microphones ($G_{1}-G_{4}$) is $\Theta =\{\theta_{1,l},\theta_{2,l},\ldots ,\theta_{4,l}|l=1,2,\ldots ,n\}$, where $\theta_{g,l}$ represents the DOA estimation value of the *l*-th sound source corresponding to the *g*-th group of microphones. The initial spatial entropy $H_{0}$ is defined as:

(15) $$H_{0}=-\sum_{s=1}^{n{!}^{4}}P\left(s\right)\log P\left(s\right),$$

where $n{!}^{4}$ refers to all possible results of selecting *n* sound source–corresponding DOAs from 4 groups of microphone DOAs (each group contains *n* DOAs); P(s) is the probability that the *s*-th combination corresponds to a real sound source. In the initial state, all combinations have equal probabilities ($P\left(s\right)=1/n{!}^{4}$), so $H_{0}=4\log n!$, and the entropy value increases sharply with the number of sound sources *n*. This model directly reflects the high uncertainty caused by the “DOA combination explosion” in multi-sound-source scenarios.

### 2.3. First-Order Entropy Reduction Based on DOA Sorting: Constraining the Combination Space

Suppose there are *n* speakers, let $\theta_{g,l}$ denote the *l*-th DOA of the *g*-th group of microphones, where g=1,2,3,4 (four groups of microphones) and l=1,2,…,n (each group has *n* DOAs). The DOAs of each group of microphones are sorted in ascending order,

(16) $$\theta_{g,1}\leq \theta_{g,2}\leq \cdots \leq \theta_{g,n},\;\mathrm{for}\;\mathrm{all}\;g=1,2,3,4.$$

We define a selection rule where, during each selection, an index *l* (l=1,2,…,n) is chosen, resulting in a total of *n* selection outcomes. The selection outcomes can be represented as

(17) $$D=\left\{(\theta_{1,l},\theta_{2,l},\theta_{3,l},\theta_{4,l})|l=1,2,\ldots ,n\right\},$$

with |D|=n, which is equal to the number of speakers. In this case, there is only one possible result for the combination of DOAs.

**Figure 2:**
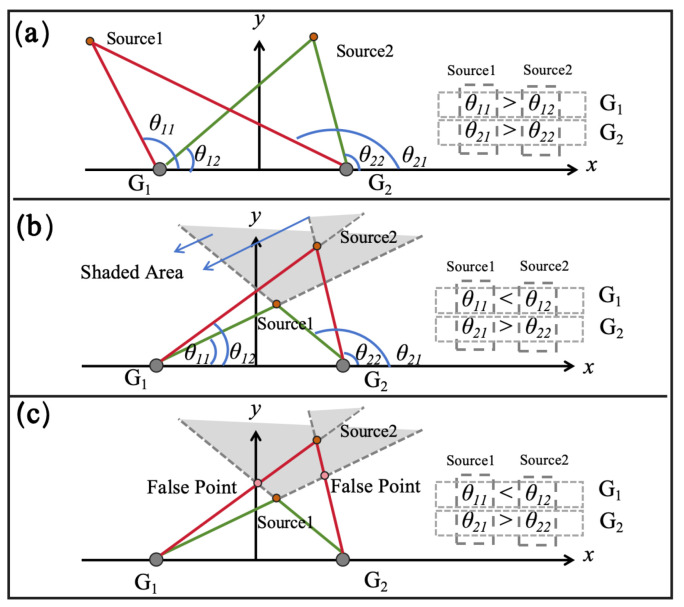
Method for multi-source localization. (**a**) After sorting the DOAs of each microphone group by magnitude, the DOA order for the same sound source is consistent across all microphone groups. (**b**) Schematic of the “Shadow Area.” If a sound source falls into the “Shadow Area,” the DOA order for the sound source in each microphone group will be disrupted. (**c**) If a sound source falls into the “Shadow Area,” “False Points” will be generated.

The selection rule we defined is based on the characteristics of our microphone array. We observe that, in the case of multiple sound sources, the order of DOA magnitudes for a sound source remains consistent across different groups of microphones. In most cases, this ensures that the selected DOAs correspond to the same sound source (Figure 2a). However, there are exceptions.

The extension line of the connection between the sound source and the microphone forms a special region, which we call the “Shadow Area”. For clarity, this is illustrated in a two-dimensional diagram (Figure 2b). When a sound source lies within the “Shadow Area” of another sound source, the order of DOA magnitudes in one group of microphones may change relative to another group. Unfortunately, we cannot detect or avoid this situation through measurements. If we proceed with our selection method to calculate the sound source location under such conditions, “False Points” may be obtained (Figure 2c).

We assume that the positions of sound sources have two states. One is that no sound source enters the “Shaded Area” at all, in which case there is only one possible result for the combination of DOAs of the *n* sound sources. The other is that some sound sources enter the “Shaded Area”. Since it is impossible to know which sound sources have entered the “Shaded Area” at the same time, there are only $n{!}^{4}$ possible results for the DOAs of the n sound sources. For the l-th sound source $p_{l}$, its “Shaded Area” can be expressed as $S_{l}$, so the probability that no sound source enters the “Shaded Area”($P_{NSA}$) can be expressed as

(18) $$P_{NSA}=P\left(\underset{1\leq l<m\leq n}{⋂}\left(p_{l}\notin S_{m}\wedge p_{m}\notin S_{l}\right)\right).$$

If the DOA combination has a probability of $P_{NSA}$ to have only one type, and a probability of $1-P_{NSA}$ to have $n{!}^{4}$ types, then the entropy of the DOA combination results can be expressed as

(19) $$H_{1}=-\left[P_{NSA}\log P_{NSA}+\sum_{i=1}^{n{!}^{4}}\left(\frac{1-P_{NSA}}{n{!}^{4}}\log\frac{1-P_{NSA}}{n{!}^{4}}\right)\right].$$

### 2.4. Second-Order Entropy Reduction Based on Geometric Intersection

After determining the DOA combination for each sound source, we can construct a spatial entropy for each sound source. We will reduce the spatial entropy of sound source localization through the following three steps.

#### 2.4.1. Step-I

Assuming that the DOA measured by each group of microphones is $\theta_{i}$, the row vector of the matrix row where the microphone pair is located is $d_{i}$, and the center of the microphone pair is $v_{i}$, then the four groups of microphones define four cone-shaped point sets related to the sound source $A=\{A_{1},A_{2},A_{3},A_{4}\}$. Each conical surface point set can be represented as

(20) $$A_{i}=\left\{p\in R^{3}|∠\left(p-v_{i},d_{i}\right)=\theta_{i}\right\}.$$

**Figure 3:**
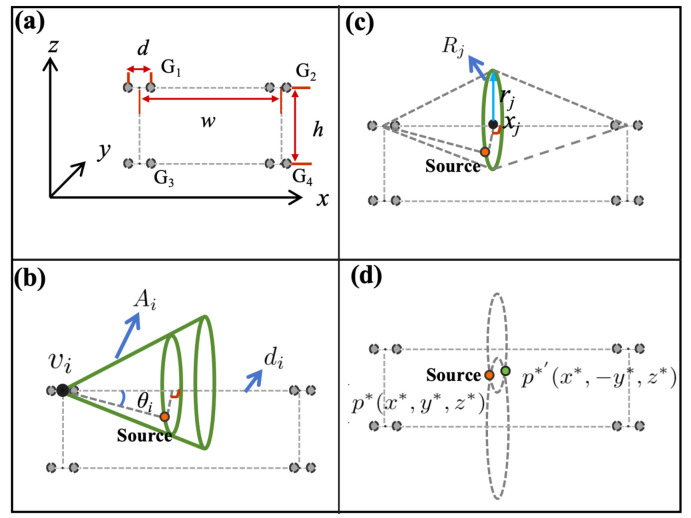
Microphone array and localization steps view. (**a**) Two by two matrix microphone array. (**b**) Step-I: Determine the point sets *A*. (**c**) Step-II: Determine the point sets *R*. (**d**) Step-III: Determine the point sets *P*.

This point set represents a conical surface with the center of the microphone pair as the vertex, the row of the matrix where the microphone pair is located as the axis of symmetry, and the generatrix forming an angle with the axis of symmetry equal to the DOA (as shown in Figure 3b). Since any point on the conical surface could potentially be the sound source, the probability P(p) is uniformly (or approximately uniformly) distributed over an infinite number of points, resulting in an extremely high entropy value:

(21) $$H_{2}=-\int_{p\in A_{i}}P\left(p\right)\log P\left(p\right)dp,$$

where P(p) is the probability density function that point *p* is the real sound source, which follows a uniform distribution.

#### 2.4.2. Step-II

As shown in Figure 3c, the intersection $R_{1}$ of the two conical surfaces $A_{1}$ and $A_{2}$ from the same microphone matrix row forms a circle, and similarly for $R_{2}$. Thus, the four conical surface point sets can determine two circular point sets related to the sound source $R=\{R_{1},R_{2}\}$, with the corresponding microphone matrix row passing through the center of the circle and perpendicular to the plane of the circle. If the radius of the circle is $r_{j}$ and the horizontal coordinate of the center is $x_{j}$, then each circular point set can be represented as

(22) $$R_{j}=\left\{p\in R^{3}|∥p-x_{j}∥=r_{j},\;d_{j}\cdot \left(p-x_{j}\right)=0\right\}.$$

Since $p^{*}$ belongs to both $R_{1}$ and $R_{2}$, $R_{1}$ and $R_{2}$ must intersect. This allows us to determine that $x^{*}=x_{1}=x_{2}$ for $p^{*}\left(x^{*},y^{*},z^{*}\right)$. In practice, we let $x^{*}=0.5\times \left(x_{1}+x_{2}\right)$. Therefore,

(23) $$R_{j}=\left\{p\in R^{3}|∥p-x^{*}∥=r_{j},\;d_{j}\cdot \left(p-x^{*}\right)=0\right\}.$$

Since any point on the circle could potentially be the sound source, the probability P(p) is uniformly (or approximately uniformly) distributed over an infinite number of points, and we can obtain $H_{3}$:

(24) $$H_{3}=-\int_{p\in R_{j}}P\left(p\right)\log P\left(p\right)dp.$$

Compared with $H_{2}$, $H_{3}$ is significantly reduced.

**Figure 4:**
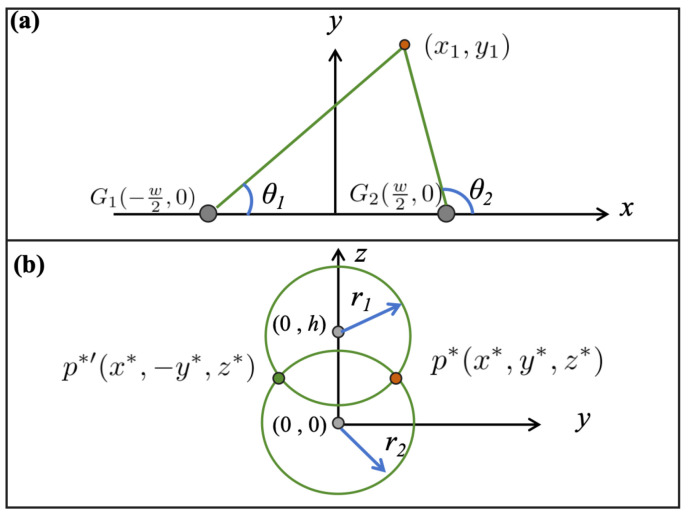
Method for solving the parameters of the point sets. (**a**) Solve the parameters $x_{j},r_{j}$ of the point set *R*. (**b**) Solve the parameters $x^{*},y^{*},z^{*}$ of the point set *P*.

Below, we provide the method for solving $r_{j}$ and $x_{j}$. For convenience, we will solve this in a two-dimensional local coordinate system (Figure 4a). We assume the center coordinates of the microphone pair are $G_{1}\left(-\frac{w}{2},0\right)$ and $G_{2}\left(\frac{w}{2},0\right)$, and the measured DOAs are $\theta_{1}$ and $\theta_{2}$. Then $x_{1}$ and $r_{1}$ can be obtained by solving the equations

(25) $$\left\{\begin{array}{c} y=\tan\left(\theta_{1}\right)\left(x+\frac{w}{2}\right),\\ y=\tan\left(\theta_{2}\right)\left(x-\frac{w}{2}\right). \end{array}\right.$$

Thus,

(26) $$x_{1}=\frac{w}{2}\cdot \frac{\tan\left(\theta_{1}\right)+\tan\left(\theta_{2}\right)}{\tan\left(\theta_{2}\right)-\tan\left(\theta_{1}\right)},$$

(27) $$r_{1}=\left|y_{1}\right|=\left|\frac{w\tan\left(\theta_{1}\right)\tan\left(\theta_{2}\right)}{\tan\left(\theta_{2}\right)-\tan\left(\theta_{1}\right)}\right|,$$

and similarly for $x_{2}$ and $r_{2}$.

#### 2.4.3. Step-III

The intersection of the two circular point sets $R=\{R_{1},R_{2}\}$ defines a point set containing two points (Figure 3d):

(28) $$P=\left\{p^{*}\left(x^{*},y^{*},z^{*}\right),\;p^{{*}^{\prime }}\left(x^{*},-y^{*},z^{*}\right)\right\},$$

where $p^{*}$ and $p^{{*}^{\prime }}$ are symmetric with respect to the plane of the microphone matrix. At this point, it can be obtained that:

(29) $$H_{4}=-P\left(p^{*}\right)\log P\left(p^{*}\right)-P\left(p^{{*}^{\prime }}\right)\log P\left(p^{{*}^{\prime }}\right).$$

Since the probabilities of the two candidate points are equal (P=0.5), $H_{4}=1$ bit.

If we restrict the working region to only one of the areas divided by the microphone matrix, we can determine a point set containing only one point:

(30) $$P^{*}=\left\{p^{*}\left(x^{*},y^{*},z^{*}\right)\right\}.$$

At this point, it can be obtained that:

(31) $$H_{5}=-log1=0.$$

At this point, we can determine the unique position of the sound source. Since Step-II provides the method for solving $x^{*}$, we now present the method for solving $y^{*}$ and $z^{*}$, also in a two-dimensional local coordinate system (see Figure 4b). Assuming the centers of $R_{1}$ and $R_{2}$ are (0,h) and (0,0), respectively, and their radii are $r_{1}$ and $r_{2}$, then $y^{*}$ and $z^{*}$ can be obtained by solving the following system of equations:

(32) $$\left\{\begin{array}{c} y^{2}+{\left(z-h\right)}^{2}=r_{1}^{2},\\ y^{2}+z^{2}=r_{2}^{2}. \end{array}\right.$$

Thus,

(33) $$y^{*}=\sqrt{r_{2}^{2}-{\left(\frac{h^{2}+r_{2}^{2}-r_{1}^{2}}{2h}\right)}^{2}},$$

(34) $$z^{*}=\frac{h^{2}+r_{2}^{2}-r_{1}^{2}}{2h}.$$

**Figure 5:**
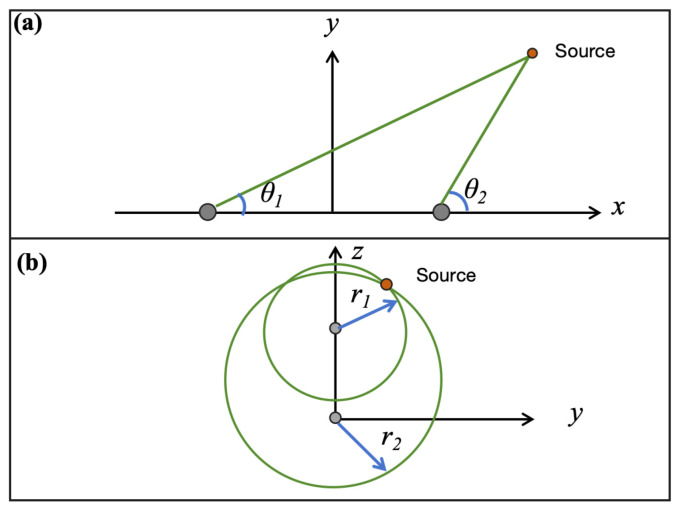
Schematic diagram of the sound source located outside the microphone array. (**a**) The $x^{*}$ is not limited to the x-axis interval of the microphone pair. (**b**) The $z^{*}$ is not limited to the z-axis interval of the microphone pair.

As shown in Formula (26), the prediction of the $x^{*}$ is mainly related to the DOA. The DOA defined by us ranges from 0 to $180^{{}^{\circ}}$, and for a pair of microphones, the range of 0–180 can cover any position. The prediction of the $y^{*}$ and $z^{*}$ is mainly related to the intersection of the two circles defined by parameters $r_{1}$ and $r_{2}$, and their intersection may be outside the microphone array. Figure 5 shows the scenario where the sound source is outside the microphone array, with the gray dots representing the microphones. In Figure 5a, the coordinate of the sound source determined by the two pairs of microphones is not limited to the x-axis interval of the microphone pairs; in Figure 5b, the $z^{*}$ of the sound source determined by the two pairs of microphones is not limited to the z-axis range of the microphone pairs.

### 2.5. Regression Model for Localization Correction

**Figure 6:**
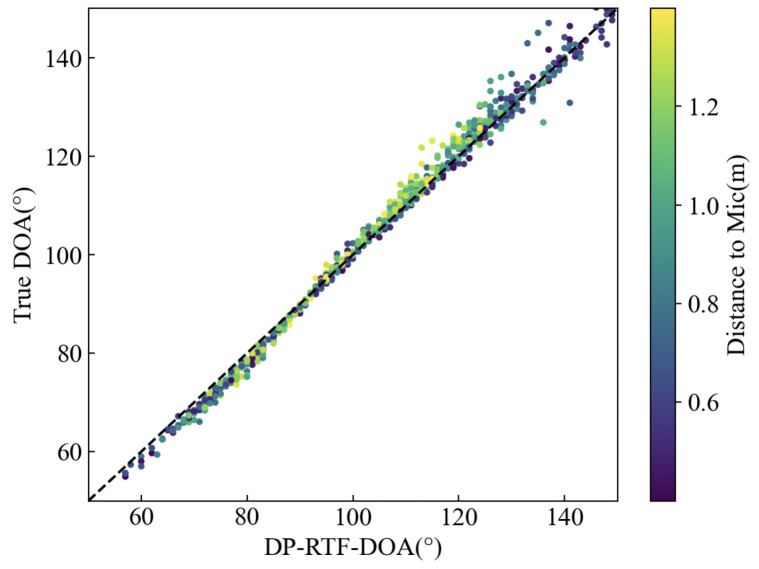
Graph of the relationship between the DP-RTF-DOA and the true DOA.

In the aforementioned method for localizing the 3D coordinates of a sound source, the precision of our approach hinges critically on the capability of each microphone array to accurately determine the DOA, which is instrumental in defining the conical surface point set *A* (Step-I). Our technique for ascertaining the DOA is grounded in the DP-RTF method [38]. Although this method can identify multiple sound sources in the presence of reverberation, it is not without its biases in DOA measurement, as illustrated in the accompanying Figure 6. Broadly speaking, the DP-RTF-DOA exhibits an incremental trend in alignment with the escalation of the true DOA. However, the true DOA corresponding to the DP-RTF-DOA is multi-valued, making it impossible to establish a direct functional mapping from DP-RTF-DOA to the true DOA. Nevertheless, when a specific distance from the microphone is given, this multi-valued phenomenon tends to diminish.

There are multiple factors contributing to the measurement errors of DP-RTF-DOA, including systematic errors, the distance between the microphone and the sound source not meeting the far-field condition, and random errors. We aim to utilize the DP-RTF-DOAs from other groups of microphones to eliminate the multi-valued phenomenon, thereby determining the parameters of the conical surface point set *A* (Step-I). In the process of solving geometric intersections, errors in DOA may accumulate and amplify, and deviations in the setting of geometric parameters can also lead to errors in the solved coordinates. To avoid such situations, we can also integrate the DP-RTF-DOAs from other groups to ascertain the parameters of the circular point set *R* (Step-II), or directly determine the parameters of the set $p^{*}$ (Step-III). We regard the DP-RTF-DOAs measured by the four groups of microphones as a set of features

(35) $$\Theta^{\prime }=\left[\theta_{1,l}^{\prime },\theta_{2,l}^{\prime },\theta_{3,l}^{\prime },\theta_{4,l}^{\prime }\right].$$

We have designed three machine learning solutions for predicting the coordinates of the sound source. The machine learning methods we employ include Random Forest (RF), Gradient Boosting Regression (GBR), Kernel Ridge Regression (KRR), Ridge Regression (RR), and Support Vector Regression(SVR).

1. In Solution-I, we aim to establish a regression model $f_{1}$ such that
  (36) $$T_{1}=f_{1}\left(\Theta^{\prime }\right),$$
  where $T_{1}=\left[\theta_{1},\theta_{2},\theta_{3},\theta_{4}\right]$ represents the true DOA, which is the parameters of the conical surface point sets *A*, and subsequently infer the true sound source coordinates $p^{*}$ in Step-II and Step-III (see Figure 1).
2. In Solution-II, we establish a regression model $f_{2}$ such that
  (37) $$T_{2}=f_{2}\left(\Theta^{\prime }\right),$$
  where $T_{2}=\left[r_{1},r_{2},x^{*}\right]$ represents the parameters of the circular point sets *R*, and subsequently infer the true sound source coordinates $p^{*}$ in Step-III (see Figure 1).
3. In Solution-III, we establish a regression model $f_{3}$ such that
  (38) $$T_{3}=f_{3}\left(\Theta^{\prime }\right),$$
  where $T_{3}=\left[x^{*},y^{*},z^{*}\right]$ represents the true sound source coordinates $p^{*}$ (see Figure 1).

## 3. Simulation Research

To amass a comprehensive dataset of acoustic sources for the construction of our model and to validate the efficacy of our eight-microphone array localization, we conducted experiments under simulated conditions utilizing Python’s Pyroomacoustics library (version 0.3.1). Pyroomacoustics is primarily employed to emulate the propagation of sound within enclosed spaces, encompassing phenomena such as reflection, absorption, and diffusion of sound waves.

### 3.1. Simulation Conditions

We set up a room with dimensions of 8 m in length, 5 m in width, and 3 m in height, adjusting the room absorption coefficient to control the reverberation time. The reverberation time (RT) refers to the time it takes for sound in a closed space to decay to 1/1000 of its original intensity after the sound source stops [44]. The reverberation time of the simulated experiment is 280 ms. The spacing between microphone pairs, denoted as *d*, is 0.2 m, while the microphone array has a width *w* of 0.6 m and a height *h* of 1 m. The coordinates of the microphone pairs and the coordinate range of the simulated sound source are shown in the Table 1. However, this configuration is not optimal, as will be discussed in the following sections. We sourced six distinct sounds from website https://freesound.org to serve as our acoustic sources (Table 2). We also add Gaussian white noise, with the signal-to-noise ratio (SNR) set at 10–15 dB.

In our simulation experiments, we gathered signals received by the microphones to compute the DP-RTF-DOAs, while also recording the coordinates of the sound sources, including the parameters of the aforementioned sets $T_{1},T_{2}$, and $T_{3}$. The speech DP-RTF signal preprocessing parameters are set as follows: the sampling rate is 16 kHz; short-time Fourier transform is used to extract time-frequency features, with a window length of 256 points, a frame shift of 128 points, and a Hamming window to reduce spectral leakage. γ is 0.2 and *Q* is 12. Calculations are performed every 0.2 s, and the candidate angles corresponding to the peak values of the mean of α across all frames are taken as the DP-RTF-DOAs samples.

However, we need to set a threshold $\alpha_{T}$ to filter out invalid peaks (i.e., false peaks caused by reverberation-induced pseudo-sound sources). A larger $\alpha_{T}$ results in fewer retained peaks, corresponding to a smaller number of candidate sound sources; conversely, a smaller $\alpha_{T}$ may fail to eliminate false peaks. To ensure the number of candidate sound sources derived from peaks matches the actual number of sound sources in the scene, it is critical to balance the value of $\alpha_{T}$.

We define the success rate (SR) as the ratio of “valid samples” to the total number of samples. Here, a “valid sample” is one where the number of candidate sound sources (determined by peaks exceeding $\alpha_{T}$) is consistent across all four microphone groups and matches the true number of sound sources in the frame. Through extensive experiments, we found that when $\alpha_{T}=0.6$, the SR reaches its maximum: 0.82 in single-source scenarios and 0.72 in multi-source scenarios.

Only these successfully detected valid samples are used to train our regression models, as they ensure the consistency of DOA combinations required for subsequent spatial entropy reduction and geometric intersection calculations. This threshold setting balances the trade-off between retaining true peaks and filtering false ones, laying a reliable foundation for accurate sound source localization. Meanwhile, in practical scenarios, we only use samples where the number of DOA peaks obtained from each microphone group is consistent as our calculation samples to determine the sound source positions. This method ensures the validity of our data, as the prerequisite for our multi-source DOA combination approach is that the number of DOA peaks from each microphone group is consistent.

### 3.2. Results of Each Solution

Different sound sources were played in space. In the single-source scenario, 658 samples were collected, among which 100 samples were used as the validation set. We used the five regression methods mentioned above to model each solution.

**Figure 7:**
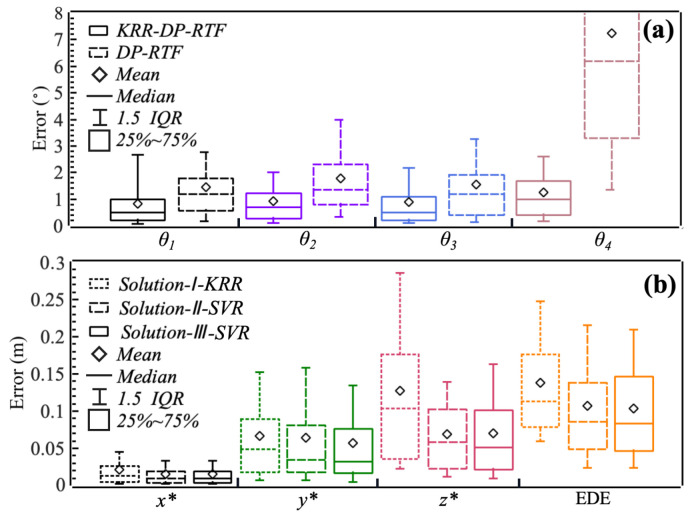
Boxplot of the localization error. The MAE of $x^{*}$, $y^{*}$, $z^{*}$ and MEDE by the Pure Geometric Method (PGM) are 0.25 m, 0.43 m, 0.51 m, and 0.65 m, respectively. (**a**) Error of the DOA ($T_{1}$) inferred by the *KRR-DP-RTF* and DP-RTF. (**b**) Error of the source coordinates $\left(x^{*},y^{*},z^{*}\right)$ inferred by *Solution-I-KRR*, *Solution-II-SVR*, and *Solution-III-SVR*.

Table 3, Table 4 and Table 5 show the $R^{2}$ of the regression models for each solution. For Solution-I, the KRR model generally performed well. KRR achieved the highest $R^{2}$ for inferring the set parameters ($T_{1}$) $\theta_{1}$, $\theta_{2}$, and $\theta_{3}$, and the $R^{2}$ for $\theta_{4}$ is also close to the optimal result of GBR. The KRR-based Solution-I (*Solution -I-KRR*) also exhibited optimal performance in inferring the source coordinates, with the highest $R^{2}$ for $x^{*}$, $y^{*}$, and $z^{*}$. However, for Solution-II and Solution-III, the best-performing model was SVR (*Solution-II-SVR* and *Solution-III-SVR*), both for inferring the set parameters ($T_{2}$ and $T_{3}$) and the source coordinates $p^{*}$. The Euclidean distance error (EDE) is defined as

(39) $$EDE=\sqrt{{\left(\Delta x\right)}^{2}+{\left(\Delta y\right)}^{2}+{\left(\Delta z\right)}^{2}},$$

where Δx, Δy, and Δz represent the errors in the inferred $x^{*}$, $y^{*}$, and $z^{*}$. We not only evaluated the mean Euclidean distance error (MEDE) between the inferred and the true sound source coordinates, but also assessed the mean absolute error (MAE) of the inferred and the true sound source coordinates along the three axes. As shown in Figure 7b, *Solution-II-SVR* and *Solution-III-SVR* outperformed *Solution-I-KRR*. The MEDE between the true source position and the inferred source position in *Solution-II-SVR* and *Solution-III-SVR* are 10.5 cm and 10.0 cm, respectively, better than *Solution-I- KRR*’s 14.0 cm. The MAE for $x^{*}$ in all three solutions is under 3.0 cm, and for $y^{*}$, it is under 8.0 cm. However, the MAE for $z^{*}$ in *Solution-II-SVR* and *Solution-III-SVR* is significantly lower than that in *Solution-I-KRR*.

**Figure 8:**
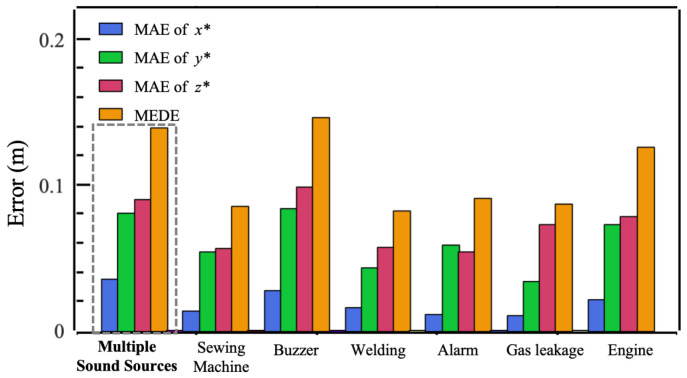
Bar chart of localization error under multiple sources and different sound sources.

In the above experiment, 657 samples were collected in the single-source scenario. Among the 100 samples used for the test set, there were 14 engine noises, 21 gas leakage sounds, 12 welding noises, 19 sewing machine sounds, 16 buzzer tones, and 18 alarm signals. It can also be seen from Figure 8 that in the single-source scenario, the model maintains good stability when inferring the positions of different types of sound sources. Except for buzzer and engine sources, which have relatively large MEDEs of 14.2 cm and 12.8 cm, respectively, the MEDEs for other source types are all below 10.0 cm. The MAE for $x^{*}$ for all source types is very small, not exceeding 4.0 cm. The position inference for the welding source type is the most accurate, with a MEDE error of 8.1 cm. The calculation of DP-RTF [38] features involves many parameters, which can be dynamically adjusted for different sound types to improve the effectiveness of the DP-RTF features. However, this is another topic and was not explored in this study.

In Solution-I, KRR performs excellently, mainly because its kernel trick can map the DP-RTF-DOA features of multiple microphone arrays to a high-dimensional space, and its L2 regularization naturally suppresses the noise interference caused by measurement errors by smoothing parameter weights. We model the DP-RTF-DOAs from multiple groups of microphones as features, aiming to correct the errors in DP-RTF-DOAs, which may stem from systematic errors, the distance between the microphones and the sound source not meeting the far-field condition, and random errors, among others. *Solution-I-KRR* is effective for correcting DOA. As shown in Figure 7a, the KRR-based DP-RTF-DOA (*KRR-DP-RTF*) generally has a smaller error than the original DP-RTF-DOA, with the average angular error reduced to $0.8^{{}^{\circ}}$–$1.2^{{}^{\circ}}$. For $\theta_{4}$, the 25–75% error range is wider, and the mean error is also large. However, the regression model can still correct it to a very small level. This indicates that our regression model can correct the systematic errors therein and is able to constrain DOA using multi-dimensional features. The approach of increasing microphone pairs to correct errors is feasible, which also points out the direction for subsequent research.

In Solution-II and Solution-III, we no longer focus on the accuracy of DOA but on solving the parameters of geometric intersections. SVR performs more prominently. In practice, no significant difference is observed in angle prediction performance between KRR, SVR, and other regression methods in Solution-I, whereas SVR outperforms other regression methods in Solution-II/III—and this discrepancy stems from the distinct task requirements of the two types of solutions. Specifically, Solution-I only requires the model to handle systematic errors in DOA measurements, while Solution-II/III impose additional demands: in addition to fitting geometric solution formulas and correcting systematic errors in geometric parameters (e.g., the width and height of the microphone array), the model must correct the sound source DOA under non-far-field conditions to achieve accurate sound source coordinates. Under far-field conditions, there is a specific mapping relationship between the sound source’s Time Difference of Arrival (TDOA, denoted as *t*) and DOA (denoted as $\theta_{DOA}$):

(40) $$\theta_{\mathrm{DOA}}=\arccos\left(\frac{t\cdot c}{d}\right),$$

where *c* represents the speed of sound and *d* denotes the distance between microphones; however, this relationship vanishes under non-far-field conditions, forcing the assumption that the DOA corresponding to the time difference obtained via DP-RTF is only approximately equal to the real DOA. Deriving the real DOA from multiple sets of approximate DOAs is challenging due to the unclear correlation between them, which is why SVR in this study adopts the Radial Basis Function (RBF) as its kernel—this kernel captures such complex relationships by mapping features to a high-dimensional space. Furthermore, geometric solution is inherently an error-accumulating process: as shown in the figures, the errors in Step-III (for solving the y and z coordinates) are larger than those in Step-II and x-coordinate solving, and abnormal DOA estimation errors are prone to amplification during geometric solution. Fortunately, the SVR in Solution II/III, which fits the geometric solution, leverages its insensitive loss function to prevent outliers from dominating model parameters, thereby ensuring superior performance.

After testing, the pure geometric method (PGM)—where the DOA of DP-RTF is used as the input for geometric intersection—has an MEDE of 68.8 cm. It can be seen from Table 3 that although $\theta_{1}-\theta_{3}$ have a certain degree of accuracy, $\theta_{4}$ has a large systematic error, leading to a relatively large overall sound source positioning error, which proves the limitations of the physical model. Solution III, a pure machine learning method, achieves high positioning accuracy but lacks a certain degree of interpretability. Solution I/Solution II are hybrid frameworks combining geometry and machine learning, which has comparable positioning accuracy compared to the pure machine learning method. Additionally, the hybrid framework allows direct observation of the parameters involved in solving the sound source coordinates ($r_{1},r_{2}$ and θ); if more feature inputs are added in subsequent studies, it will be possible to more intuitively understand how these features affect the solution of sound source positioning.

Although end-to-end models such as *Solution-III* do not explicitly invoke geometric formulas, they have implicitly encoded geometric constraints into their mapping rules through a large number of samples containing spatial geometric relationships. For instance, in *Solution-III-SVR*, the error in the x-axis is significantly lower than that in the y-axis and z-axis, which is fully consistent with the geometric principle that “the x-coordinate is less affected by DOA errors” (see Figure 7b). This rules out the randomness of the error distribution and confirms the model’s effectiveness in learning geometric constraints. Tests on the input features of the *Solution-III-SVR* model show the following (Table 6): After swapping $\theta_{1}$, $\theta_{2}$ with $\theta_{3}$, $\theta_{4}$, the model still maintains high accuracy in x-coordinate prediction, which aligns with the principle in Equation (26) that “the x-coordinate can be calculated using only the DOAs of microphones on the same x-axis”. However, when the DOAs of only one set of microphones are perturbed, the model’s x-coordinate prediction accuracy decreases significantly—this indicates that the model also balances the x-coordinate calculations from the upper and lower sets of microphones. In contrast, since the y and z coordinates depend on all four sets of DOAs, the model cannot predict them accurately when encountering the aforementioned disturbances. Despite adopting an end-to-end learning approach, the *Solution-III-SVR* model exhibits a certain degree of interpretability.

Solution-III skips geometric intersection to a certain extent, spatial entropy still holds value. First, we still use sorted DOA combinations as model inputs to ensure the input DOAs belong to the same sound source, which reflects the reduction in spatial entropy. Second, through the deduction of geometric intersection, we have confirmed that four groups of DOAs can reduce the spatial entropy of the sound source to 0, which not only provides a basis for selecting model feature inputs but also lays a foundation for the model’s interpretability. However, the relatively large solution errors of the y and z coordinates contradict the conclusion that spatial entropy is 0, and the relationship between them remains unclear. Future studies will incorporate more DOA features to explore this relationship and further improve the relevant theory.

In summary, the 3D sound source localization scheme and the regression model we designed are effective. We not only corrected the errors in DOA calculation by DP-RTF, which may arise from DP-RTF itself or the microphone pairs not meeting the far-field model assumptions, but also used regression models to eliminate systematic errors in solving geometric intersections.

### 3.3. Performance of *Solution-III-SVR* Model

From the above results, it can be seen that the source position inferred by the *Solution-III-SVR* model has the smallest error. Next, we will test its performance in different environments.

#### 3.3.1. Environments with Different Reverberation Levels

**Figure 9:**
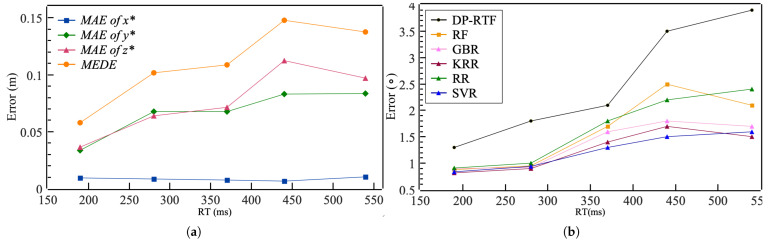
(**a**) Error of localization under different reverberation conditions. (**b**) Error of DOA under different reverberation conditions.

To test the localization accuracy of the *Solution-III-SVR* model under conditions of different reverberation levels, we adjusted the reverberation coefficient of the room. We collected 120 valid samples under the condition that $\alpha_{T}$ is 0.6. Among them, the RT is set to 190 ms, 370 ms, 440 ms, and 540 ms, with 30 samples for each RT. We can see that the model’s performance varies significantly (Figure 9a). It can be observed that as the reverberation level increases, the MAE in $y^{*}$, $z^{*}$, and the MEDE also tend to increase. This suggests that the reverberation level of the room is a major factor affecting the model’s localization performance. Under weak reverberation conditions (RT = 190 ms), the MEDE can even reach 5.8 cm. However, the reverberation level does not have a significant effect on the error of $x^{*}$.

We also measured the DP-RTF-DOAs and tested the correction effect of model I on them to find out how reverberation affects localization accuracy. It can be observed that as the reverberation level increases, the error of DP-RTF-DOAs also increases. As shown in Figure 9b), the model in Solution-I has a certain correction ability for it. However, as the reverberation level increases, the error will also increase accordingly.

Our model was sampled and trained under the condition of an RT of 280 ms, yet it exhibits smaller localization errors in scenarios with an RT of 190 ms. This indicates that our model can effectively capture the relationship between the multi-group DOA features and the sound source position. However, strong reverberation will increase the random errors in DOA measurement, which is reflected in the sharp rise of the MEDE when the RT is 440 ms and 540 ms. This is also reflected in the fact that, in the model of Solution-I, the error of DOA prediction increases with the rise in reverberation level. Nevertheless, the prediction error of the $x^{*}$ does not show a tendency to increase with the enhancement of reverberation, maintaining a relatively consistent error across all reverberation levels. This implies that the random errors caused by reverberation can be eliminated through the DOA features captured by multiple groups of microphones. In the process of solving geometric intersections, the $x^{*}$ is inferred prior to the $y^{*}$ and $z^{*}$, which prevents the accumulation and amplification of DOA errors on the $x^{*}$. This may also be the reason why the localization error of the $x^{*}$ remains consistent under different reverberation levels. The shape of our microphone array determines the inference order of the $x^{*}$, $y^{*}$, and $z^{*}$, which provides a direction for future research: can we also improve the localization accuracy of the $y^{*}$ and $z^{*}$ by changing the shape of the microphone array, making their inference process the same as that of the $x^{*}$?

#### 3.3.2. Results of Different Microphone Configurations

The above results show that the performance of *Solution-III-SVR* is the best, but this is only based on a fixed microphone configuration (the height *h* and width *w* of the microphone array). This section will explore how the microphone configuration affects the model’s performance. In the simulated scenario, we will change the *h* and *w* of the microphone array. Due to the changes in the microphone configuration, new samples must be collected and the model retrained for each configuration, followed by performance testing. We conducted two sets of experiments. One set fixed the *h* of the microphone array at 2.0 m and varied the *w* to investigate how the *w* affects the detection distance and model accuracy. The other set fixed the *w* of the microphone array at 0.6 m and varied the *h* to explore how the *h* affects the detection distance and model accuracy. In each scenario, we collected 800 valid samples under the condition that $\alpha_{T}$ is 0.6. To explore the performance of the model at different detection distances, we also divided the data according to the distance from the microphone. Only those samples that are less than the detection distance will be used for training.

**Figure 10:**
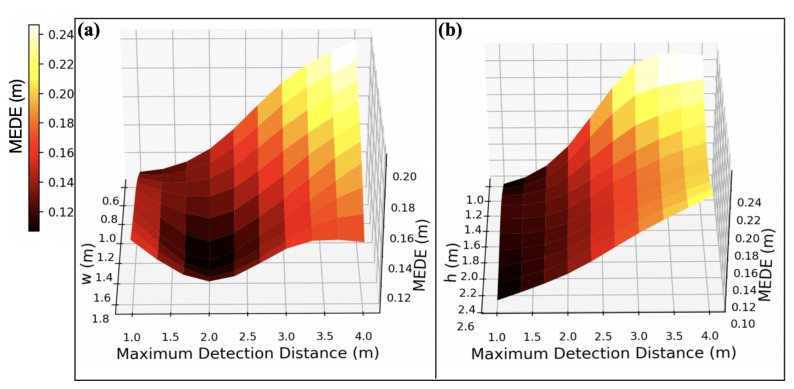
The relationship between microphone configuration and localization error. (**a**) MEDE of localization under a given microphone array w and maximum detection distance. (**b**) MEDE of localization under a given microphone array h and maximum detection distance.

From Figure 10, it can be seen that, under fixed scenarios, the microphone configuration significantly impacts its performance. In the first set of experiments, it can be observed that when the *w* is less than 1.0 m, the detection accuracy decreases significantly as the detection distance increases. As the *w* increases, the accuracy of short-range detection (less than 1.5 m) slightly decreases, but the accuracy of long-range detection (2.0–4.0 m) drops significantly. When the *h* is 1.75 m, the accuracy is optimal for maximum detection distances of 2.0 m and greater than 3.0 m. In the second set of experiments, regardless of the *h* variation, the accuracy for all detection distances did not show significant changes. Moreover, the accuracy decreased significantly as the detection distance increased. This may be due to the fixed *w* of 0.6 m being too small. However, we have not yet tested other combinations of *h* and *w* and their impact on detection accuracy, which will be explored in future studies.

We found that when constructing the model, the width *w* has a certain impact on the accuracy of the model’s positioning at different distances. As can be seen from Equations (26) and (27), the width *w* mainly affects the calculation results of $x^{*}$ and *r*. Starting from these two equations, we will simulate the impact of a $0.5^{{}^{\circ}}$ DOA deviation on $x^{*}$ and *r* under specific sound source distances and specific width *w*.

**Figure 11:**
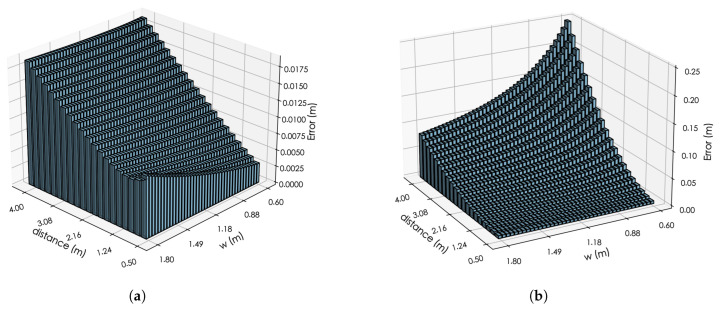
(**a**) The impact of a $0.5^{{}^{\circ}}$ DOA error on $x^{*}$ under different distances and *w* conditions. (**b**) The impact of a $0.5^{{}^{\circ}}$ DOA error on *r* under different distances and *w* conditions.

It can be seen from Figure 11a that as the sound source distance increases, the error of the $x^{*}$ gradually increases. Additionally, as the width *w* increases, the error of the $x^{*}$ also gradually increases, but this trend is not very obvious. Moreover, the error of $x^{*}$ remains at a very small value, which is also consistent with the previous conclusion.

As shown in Figure 11b, the error of *r* gradually increases with the increase in the sound source distance. However, when the width *w* is very small, the error of *r* at long distances will be extremely large. As the width *w* gradually increases, the error of *r* at long distances decreases significantly. Compared with the error of $x^{*}$ (which is less than 2.0 cm), the error of *r* can reach the level of 10.0 cm.

Therefore, it can be concluded that *w* mainly affects the calculation of the long-distance *r* parameter, thereby affecting the calculation of the $y^{*}$ and $z^{*}$. However, increasing the width *w* can reduce the estimation error of the long-distance *r* parameter. This result is consistent with the model results in Figure 10: as the width *w* increases, the model’s positioning accuracy for long distances improves. Nevertheless, when the width gradually increases, the model’s positioning accuracy for short distances shows a slight upward trend. This may be attributed to the insufficient number of short-distance samples during model training, which prevents the model from effectively extracting the relationship between features and coordinates. Another possible reason is the increased error of the $x^{*}$ caused by the increase in the width *w* (as shown in Figure 11a).

#### 3.3.3. Results of Different DOA Algorithms

**Figure 12:**
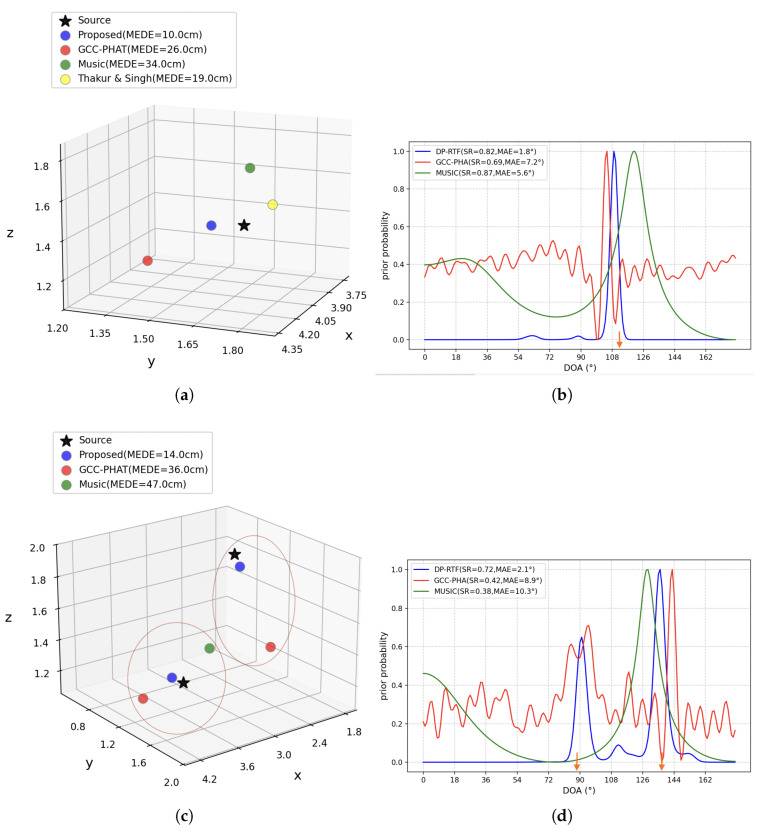
Comparison of positioning performance among different methods: (**a**) An example of the positioning performance of different methods in a single sound source scenario. (**b**) An example of the prior probabilities calculated by different DOA algorithms in a single sound source scenario, where the true DOA is $112^{{}^{\circ}}$ (the angle pointed to by the orange arrow). (**c**) An example of the positioning performance of different methods in a two-sound-source scenario, where the red circles indicate the positioning results for the same sound source. (**d**) An example of the prior probabilities calculated by different DOA algorithms in a two-sound-source scenario, where the true DOAs are $88^{{}^{\circ}}$ and $136^{{}^{\circ}}$ (the angles pointed to by the orange arrow).

We also tested the performance of 3D positioning using DOAs obtained by GCC-PHAT and MUSIC, and tested the positioning performance of Thakur & Singh’s [34] method. Figure 12 shows an example of the DOAs and 3D coordinates calculated by these methods in single-source and a two-sound-source scenario, respectively. We simulated 300 pieces of data, respectively, for the single-sound-source and two-sound-source scenarios, which are used for subsequent testing.

As shown in Figure 12b,d, the DOA prior probabilities are calculated by a set of microphones, similar to DP-RTF; GCC-PHAT and MUSIC calculate the prior probability for each candidate DOA when obtaining DOAs. The DOA and the number of sound sources are determined based on the peaks. Similarly, $\alpha_{T}$ need to be set for GCC-PHAT and MUSIC to filter out invalid peaks. Through experiments, it is found that when the $\alpha_{T}$ for GCC-PHAT and MUSIC are set to 0.7 and 0.5, respectively, the SR reaches its highest value. We found that in the single-source and the two-sound-source scenario, the SR values of the proposed method (which uses DP-RTF to calculate DOA), GCC-PHAT, and MUSIC are different. In the single-source scenario, all three methods achieve a relatively high SR, with MUSIC having the highest SR, followed by the proposed method. In the two-source scenarios, the SR of all three methods decreases. The proposed method maintains a relatively high level of 0.73, while GCC-PHAT and MUSIC decrease significantly to 0.42 and 0.38, respectively.

In the single-source example shown in Figure 12b, the true DOA of the sound source is $112^{{}^{\circ}}$. All three DOA algorithms yield distinct peaks: $109^{{}^{\circ}}$ for DP-RTF, $105^{{}^{\circ}}$ for GCC-PHAT, and $120^{{}^{\circ}}$ for MUSIC. These results all deviate somewhat from the true value of $112^{{}^{\circ}}$, which explains why GCC-PHAT and MUSIC exhibit larger positioning errors (Figure 12a). However, since DP-RTF undergoes processing by a regression model, its positioning error is relatively small. In the multi-source example shown in Figure 12d, the true DOAs of the sound sources are $88^{{}^{\circ}}$ and $136^{{}^{\circ}}$. Based on the detected peaks, the results are as follows: DP-RTF yields $90^{{}^{\circ}}$ and $135^{{}^{\circ}}$; GCC-PHAT yields $94^{{}^{\circ}}$ and $143^{{}^{\circ}}$; and MUSIC yields $129^{{}^{\circ}}$. Among these, the results from DP-RTF and GCC-PHAT are valid samples, while those from MUSIC are invalid because the number of their candidate sound sources is inconsistent with the actual number of sound sources.

We found that when moving from a single-sound-source scenario to a two-sound-source scenario, the SR and MAE of all three DOA algorithms degrade, but DP-RTF performs relatively well. MUSIC has a high SR in the single-sound-source scenario but drops sharply in the two-sound-source scenario. This may be because the algorithm can only detect one sound source in multi-source scenarios, which can also be seen in Figure 12d. Although the MUSIC algorithm is applicable to multi-source scenarios, it requires the number of sensors to be greater than that of sound sources to ensure effective separation of subspaces. In this study, only two microphones were used in each group, and in the two-sound-source scenario, the insufficient number of sensors violated the applicable conditions of the algorithm, resulting in performance degradation. The SR of GCC-PHAT also decreases in the two-sound-source scenario, which may be attributed to its poor handling of reverberation. It can be observed that in the single-sound-source case, GCC-PHAT detects many spurious peaks. However, these peaks are small and do not exceed the $\alpha_{T}$, so its SR can still remain at a relatively good level. However, in the two-sound-source scenario, the spurious peaks are strengthened; if they exceed the $\alpha_{T}$, the number of candidate sound sources will increase, leading to a decrease in SR.

The SSL of our microphone array relies on DOA. Therefore, the DOAs calculated by GCC-PHAT and MUSIC can also be used for SSL. We also tested Thakur & Singh’s 3D SSL, which uses a conical five-microphone array to perform 3D localization based on the time delay information of microphone pairs calculated by the GCC-PHAT and the energy difference of signals between microphone pairs. However, this method is not applicable in multi-source scenarios.

As shown in Figure 12a,c, in the single-source scenario, the proposed method has the lowest MEDE, followed by Thakur & Singh’s method. GCC-PHAT and MUSIC do not perform well on our microphone array. This is attributed to the large errors in its DOA measurements. The same applies to GCC-PHAT. The error (19.0 cm) of Thakur & Singh’s method is smaller than that of our method using GCC-PHAT (26.0 cm). This may be attributed to the fact that Thakur & Singh’s method also uses the energy difference of signals between microphone pairs or the characteristics of their microphone array geometry. In the two-source scenario, the proposed method still shows good performance. However, there is still an increase in MEDE, which may be due to the increased random errors in DP-RTF-DOA measurements in multi-sound-source scenarios. Since the MUSIC sample detected only one DOA, i.e., one sound source, this sample is invalid. Even though this sound source seems to belong to the one on the left in Figure 12c, we detected only one peak, making it impossible to determine which one it belongs to.

In addition to high-precision localization, compared with the methods (Table 7) of Thakur & Singh [34], Li et al. [21], Yang et al. [36], Wang et al. [10], Luo et al. [11], and Dehghan Firoozabadi et al. [51], the SSL method we proposed has certain robustness under reverberation. Compared with the methods of Thakur & Singh [34], Krause et al. [33], Lee & Kim [21], Yang et al. [36], Wang et al. [10], and Luo et al. [11], our method can work in multi-source scenarios. Meanwhile, our method uses eight microphones, maintaining a relatively low computational load and complexity. Compared with the method of Dehghan Firoozabadi et al. [51], although it can also achieve multi-source localization, it requires distributing 38 microphones in the room and then mobilizing different microphone groups according to the positions of different sound sources, which is relatively complex.

## 4. A Practical Case of Our Method

**Figure 13:**
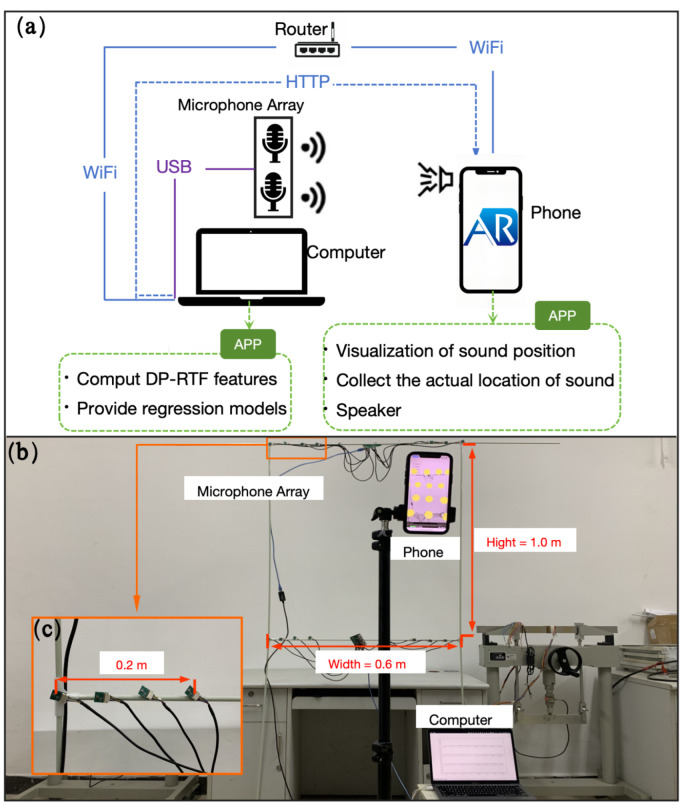
Practical case view. (**a**) Schematic diagram of the composition framework of the practical case. (**b**) Real component image of the practical case. (**c**) Image of the microphone pair.

We built an SSL system to validate the effectiveness of our proposed method. The system framework is shown in Figure 13a. The *w* of the microphone array is 0.6 m, the *h* is 1.0 m, and the distance between microphone pairs is 0.2 m (Figure 13b,c). The microphone array is connected to the computer via a USB cable. We chose a relatively empty classroom as the experimental site, which is 8 m long, 8 m wide, and 3 m high. The speech DP-RTF signal preprocessing parameters are set as follows: the sampling rate is 16 kHz; short-time Fourier transform is used to extract time-frequency features, with a window length of 256 points, a frame shift of 128 points, and a Hamming window to reduce spectral leakage; and the γ and *Q* are 0.2 and 12. The coordinates of the microphone pairs and the coordinate range of the simulated sound source are shown in Table 8.

The computer acts as a server, processing the data collected by the microphones, mainly to calculate the DP-RTF-DOA. Additionally, the computer stores a pre-built regression model that can directly infer the sound source coordinates after the DP-RTF-DOAs are calculated. However, the model built in the simulated scenario cannot be directly applied to the real-world scenario, because many factors in the actual environment, such as the room’s sound absorption coefficient and microphone device characteristics, are unknown. We also need to collect samples in the real-world scenario and rebuild the model. However, it is difficult to accurately capture the sound source position in the real-world scenario

The system can achieve real-time operation, and its real-time performance mainly depends on the complexity of the DP-RTF calculation and the regression model. In the DP-RTF calculation, the CTF length (Q = 12, corresponding to 0.55 s reverberation) determines the dimension of vector $z_{p,k}$ (2Q) and the scale of matrix ${\hat{\Phi }}_{zy}^{s}\left(p,k\right)$ (O×2Q). The number of frequency points (K) is reduced in redundant calculations by cropping the voice-active frequency band (300–1000 Hz). The number of candidate positions (S = 181, covering 0–$180^{{}^{\circ}})$) balances spatial resolution and clustering time. The complexity of the SVR regression model is dominated by the number of support vectors (571). We define the Real-Time Factor (RTF) as

(41) $$RTF=\frac{t_{p}}{t_{s}},$$

where $t_{p}$ is the system processing time and $t_{s}$ is the actual signal duration. Experiments were conducted using four groups of eight-microphone arrays (16 kHz sampling rate), and the real-time factor RTF on the Apple M1 processor (manufacturer: Apple Inc., city: Cupertino, country: United States; 8 GB memory, no GPU acceleration) was 0.72 (RTF < 1), verifying the real-time performance.

We developed an iOS application based on Apple’s ARKit. The app builds an AR space with the phone as the observer, which allows real-time tracking of the phone’s position in the AR space. It also enables the visualization of any given coordinate (e.g., a sound source) in the AR space. Not only is the phone used as an observer, but it is also used as the speaker, so the sound source’s coordinates correspond to the phone’s coordinates. The computer and phone are connected through a router, and the computer transmits data to the phone via HTTP. During the sample collection phase, the phone acts as the speaker and emits sound, while the computer transmits the processed microphone DP-RTF-DOA to the phone. At the same time, the phone saves its own position and the DP-RTF-DOA as a sample.

The collection process is as follows: A location is selected in the classroom, and the above-mentioned six types of sounds are played randomly, each lasting 3 s. The DP-RTF-DOA calculation is performed every 0.2 s. With $\alpha_{T}$ set to 0.6, all valid DOAs within the 3 s are collected, and their average value is taken as a sample. Meanwhile, the real coordinates of the sound source are recorded. The above operations are repeated after changing the location. Finally, a total of 257 samples are collected. In total, 230 samples were used to train the regression model, and 27 samples were used for testing. To collect test samples in dense sound source scenarios, we placed two or three speakers in the scene as sound sources. We had these speakers emit sound simultaneously and recorded the DOA features of DP-RTF. Then, we moved the AR device to the positions of these sound sources to record their coordinates. In both the two-source and three-source scenarios, we collected 20 samples, respectively.

**Figure 14:**
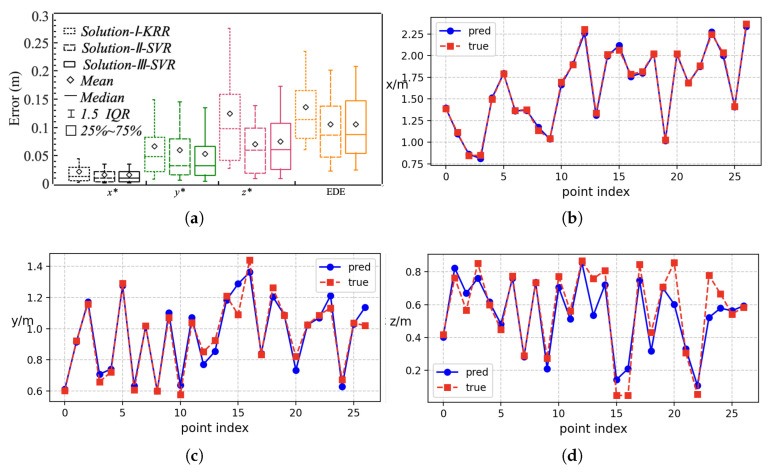
The results of the test samples: (**a**) Error of the source coordinates inferred by *Solution-I-KRR*, *Solution-II-SVR*, and *Solution-III-SVR*. (**b**) The true values and predicted values of the *x*-coordinates in the test set of the *Solution-II-SVR* model. (**c**) The true values and predicted values of the *y*-coordinates in the test set of the *Solution-II-SVR* model. (**d**) The true values and predicted values of the *z*-coordinates in the test set of the *Solution-II-SVR* model.

**Figure 15:**
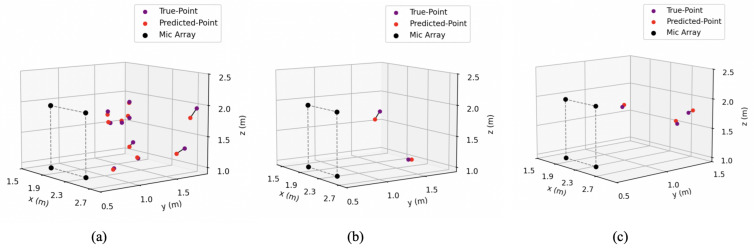
Visualization of sound source localization. The rectangle formed by dashed lines is the microphone array. (**a**) The true and predicted positions of sound sources in some single-source scenarios. (**b**) An example of true and predicted positions in a 2-source scenario. (**c**) An example of true and predicted positions in a 3-source scenario.

As in the previous simulation experiments, we also used three solutions, each employing five regression methods. As shown in Figure 14 and Figure 15a, our SSL method exhibits excellent accuracy. The MEDEs for *Solution-II-SVR* and *Solution-III-SVR* are 10.2 cm and 10.5 cm, respectively, which are close to the simulation results (10.5 cm and 10.0 cm) and significantly lower than *Solution-I-KRR* (13.5 cm). The primary reason for the higher MEDE in *Solution-I-KRR* is the large error in $z^{*}$, which is consistent with the simulation experiment. The errors for $x^{*}$ in all three solutions are similarly low, not exceeding 3.0 cm. Experimental results show that the Mean Euclidean Distance Error (MEDE) is 12.2 cm in the two-source scenario and 13.7 cm in the three-source scenario, maintaining a certain level of localization accuracy. Even when multiple sound sources are placed relatively close to each other (with a spacing of less than 1.5 m), the proposed method can still effectively distinguish their positions (Figure 15b,c).

We have demonstrated the feasibility of our 3D sound source localization method through practical examples, and the model we constructed shows the same trend as the simulation experiments. SVR yields good fitting results in *Solution-II* and *Solution-III*. As can be seen from Figure 14b–d, in the test set, the coordinate values predicted by the model are highly consistent with the real coordinates. The predicted values of the x-coordinate are almost identical to the real values, which is also consistent with the previous conclusion.

## 5. Conclusions

According to previous studies, SSL has been widely used in various applications. To address the challenge of performing three-dimensional sound source localization in scenarios with multiple sound sources, we designed a sound source localization method using an eight-microphone rectangular array. We employ a DOA algorithm based on DP-RTF and geometric intersection solving to achieve three-dimensional sound source localization, and we propose solutions for multiple sound source scenarios based on the shape of the microphone array. Furthermore, this study quantifies the uncertainty caused by the explosion of DOA combinations in multi-source scenarios by introducing spatial entropy, and realizes the stepwise reduction in spatial entropy through the DOA sorting and geometric intersection strategies of the microphone array. To resolve the instability of DOA calculations using DP-RTF and correct deviations in microphone geometric parameters, we also use machine learning to correct errors. The experimental results demonstrate that the proposed eight-microphone rectangular array method can accurately localize sound sources in three-dimensional space, achieving a MEDE of the order of 10.0 cm. Additionally, our machine learning approach, which combines DOA features from multiple microphones, effectively reduces systematic errors, random errors, and errors caused by non-far-field models in DOA estimation using DP-RTF. Our microphone array can estimate the *x*-coordinate with an accuracy of 2.0 cm under different reverberation conditions and various microphone configurations. Future work aims to further improve the localization accuracy of the *y* and *z* coordinates to match the level of the *x*-coordinate.

## Figures and Tables

**Table 1:** Microphone pair coordinates and simulated sound source position.

	x/m	y/m	z/m
$G_{1}$	3.6	0.6	1.0
$G_{2}$	4.2	0.6	1.0
$G_{3}$	3.6	0.6	2.0
$G_{4}$	4.2	0.6	2.0
Sound	2.5–6.5	0.5–4.0	0.5–2.2

**Table 2:** Types of sound sources and number of collected samples in the simulation experiment.

Name	Description	Number of Samples
Engine noises [45]	Car engine being started, run idle then stopped.	112
Gas leakage sound [46]	Hissing leaking gas recorded by zoom h2.	107
Welding noises [47]	Noise of a welding machine.	114
Sewing machine sounds [48]	Sound of an old sewing machine/sound of an old sewing machine.	98
Buzzer tones [49]	A weird sensor alarm that talks and beeps at you.	113
Alarm signals [50]	Alarm clock sound effect recorded in ableton live.	104

**Table 3:** $R^{2}$ of the Solution-I model in the simulation experiment. The bold values in the table represent the best results.

Method	$T_{1}$				$p^{*}$		
	$\theta_{1}$	$\theta_{2}$	$\theta_{3}$	$\theta_{4}$	$x^{*}$	$y^{*}$	$z^{*}$
RF	0.993	0.989	0.993	0.993	0.983	0.856	0.849
GBR	0.995	0.996	0.996	**0.996**	0.983	0.858	0.829
KRR	**0.997**	**0.997**	**0.997**	0.994	**0.985**	**0.895**	**0.882**
RR	0.993	0.995	0.993	0.989	0.982	0.804	0.761
SVR	0.995	0.996	0.995	0.991	**0.985**	0.833	0.751
PGM	0.991	0.988	0.989	0.701	0.811	0.691	0.688

**Table 4:** $R^{2}$ of the Solution-II model in the simulation experiment. The bold values in the table represent the best results.

Method	$T_{2}$			$p^{*}$	
	$r_{1}$	$r_{2}$	$x^{*}$	$y^{*}$	$z^{*}$
RF	0.846	0.855	0.996	0.792	0.844
GBR	0.872	0.889	0.995	0.835	0.871
KRR	0.879	0.859	0.993	0.830	0.838
RR	0.669	0.614	0.961	0.457	0.581
SVR	**0.905**	**0.914**	**0.997**	**0.870**	**0.939**

**Table 5:** $R^{2}$ of the Solution-III model in the simulation experiment. The bold values in the table represent the best results.

Method	$T_{3}$ ($p^{*}$)		
	$x^{*}$	$y^{*}$	$z^{*}$
RF	0.996	0.853	0.840
GBR	0.995	0.846	0.758
KRR	0.993	0.895	0.813
RR	0.961	0.562	0.484
SVR	**0.997**	**0.894**	**0.934**

**Table 6:** MAE comparison of different input combinations in simulation experiments.

Input Combination	MAE (m)		
	x	y	z
$\theta_{1}$, $\theta_{2}$, $\theta_{3}$, $\theta_{4}$	0.02	0.08	0.09
$\theta_{1}$, $\theta_{2}$, $\theta_{1}$, $\theta_{2}$	0.04	0.27	0.41
$\theta_{3}$, $\theta_{4}$, $\theta_{3}$, $\theta_{4}$	0.06	0.31	0.49
$\theta_{1}$, $\theta_{2}$, $\theta_{1}$, ⊗	0.41	2.27	1.15

**Table 7:** Comparison of the method in this study with other methods.

Method	2D/3D	MEDE	Applicable to Reverberation	Applicable to Multiple Sound Sources	Number of Microphones
Thakur & Singh [34]	3D	19 cm	No	No	5
Li et al. [38]	2D	/	Yes	Yes	2
Krause et al. [33]	2D	1.6 m	Yes	No	2
Lee & Kim [21]	3D	3 cm	No	No	5
Yang el al. [36]	3D	7 cm	No	No	7
Wang et al. [10]	2D	10 cm	No	No	/
Luo et al. [11]	2D	/	No	No	8
Dehghan Firoozabadi et al. [51]	3D	30–40 cm	No	Yes	38
Proposed	3D	5–15 cm	Yes	Yes	8

**Table 8:** The coordinates of the microphones and the coordinates of the sound sources in our case.

	x/m	y/m	z/m
$G_{1}$	1.6	0.6	1.0
$G_{2}$	2.2	0.6	1.0
$G_{3}$	1.6	0.6	2.0
$G_{4}$	2.2	0.6	2.0
Sound	0.6–3.0	0.5–2.0	0.6–2.1

## Data Availability

Dataset available on request from the authors.

## Abbreviations

The following abbreviations are used in this article:

SSL	Sound Source Localization
DOA	Direction Of Arrival
GCC	Generalized Cross-Correlation
MUSIC	Multiple Signal Classification
GCC-PHAT	Generalized Cross-Correlation–Phase Transform
DP-RTF	Direct Path Relative Transfer Function
RF	Random Forest
GBR	Gradient Boosting Regression
KRR	Kernel Ridge Regression
RR	Ridge Regression
SVR	Support Vector Regression
RT	Reverberation Time
EDE	Euclidean Distance Error
SR	Success Rate
MEDE	Mean Euclidean Distance Error
MAE	Mean Absolute Error
Solution-I-KRR	Solution-I based on KRR
Solution-II-SVR	Solution-II based on SVR
Solution-III-SVR	Solution-III based on SVR
